# Passive Exoskeleton-Assisted Gait Shows a Unique Interlimb Coordination Signature Without Restricting Regular Walking

**DOI:** 10.3389/fphys.2022.916185

**Published:** 2022-06-13

**Authors:** Takashi Sado, Zachary Motz, Jennifer M. Yentes, Mukul Mukherjee

**Affiliations:** ^1^ Department of Biomechanics, University of Nebraska at Omaha, Omaha, NE, United States; ^2^ Department of Health & Kinesiology, Texas A&M University, College Station, TX, United States

**Keywords:** NonLinear Analysis, Walking, Motor Control, Cross Recurrence Quantification Analysis, Cross sample entropy

## Abstract

Exoskeleton assistive devices have been developed as a potential approach to solve gait deficits like paretic propulsion and reduced speed. However, it is unclear how these devices affect inter-limb coordination. The duration and the synchrony of gait coordination was assessed during passive exoskeleton-assisted walking in healthy young individuals. It was hypothesized that inter-limb coordination would be reduced in comparison to normal walking without assistance, thus demonstrating gait with exoskeleton to be more explorative and flexible. Eighteen participants were divided into two groups (EXO: *n* = 9; NO EXO: *n* = 9) and performed a 5-min walking trial at a preferred walking speed after a familiarization trial. The duration of inter-limb coordination was examined using cross-recurrence quantification analysis and the synchrony was measured using cross sample entropy. There were no significant differences in spatiotemporal measurements between the two groups. However, in comparison to the no exoskeleton group, there was a reduction in the duration of coordination (mean diagonal length: *p* < 0.01) and the synchrony of coordination (entropy value: *p* < 0.05) in the exoskeleton group. These results indicate that exoskeletal-assisted gait is characterized by reduced inter-limb coordination possibly for allowing gait patterns to be more explorative and flexible. This is important in rehabilitation of patients who suffer from coordination deficits.

## 1 Introduction

### 1.1 A Theoretical Model of Inter-Limb Coordination

Inter-limb coordination is a term that characterizes the movement of one limb in relation to another during specific tasks such as walking. In a healthy human, this coordination is smooth and shows flexibility to different tasks and environments. Inter-limb coordination is affected by the neural linkage between limbs and is flexible in a way that can be adjusted to different tasks and environments (Dietz and Schrafl-Altermatt, 2016). Two legs can be coordinated perfectly as in a robotic walking system (Collins et al., 2015), or poorly coordinated, resulting from random movements as in ataxic gait. Neither of these demonstrate optimal coordination, a hallmark of healthy natural gait. This is because when stressful environments or tasks are encountered (e.g. sideways perturbation), both of these coordination patterns generally fail, leading to falls.

**FIGURE 1:**
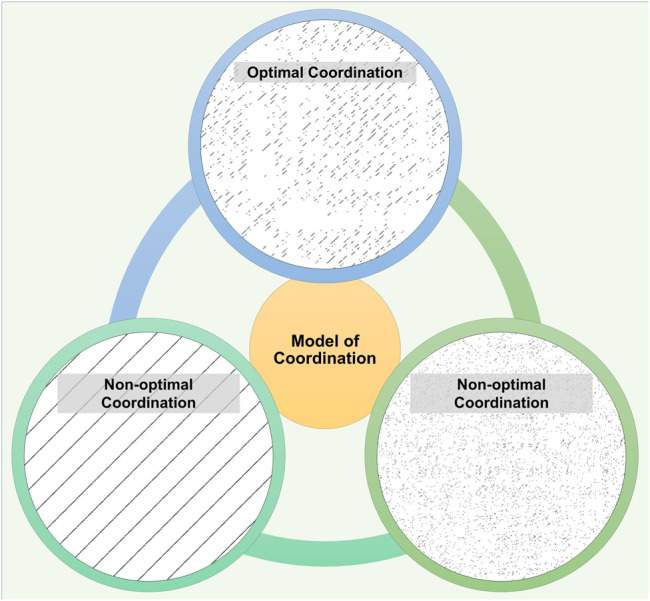
The model of inter-limb coordination. In this model, healthy inter-limb coordination sits at a sweet-spot between patterns that exhibit either metronomic qualities or random-like qualities. Healthy individuals demonstrate optimal coordination (top) where the cross recurrence plot shows an intermediate level of diagonal lines (representative recurrence plot from the control group). Diagonal lines indicate the length of time that the right and left limbs are coordinated. Longer or shorter diagonal lines indicate a non-optimal coordinated behavior (bottom left and right). Bottom left shows the recurrence plot with two simulated sinusoidal time series and long diagonal lines, as would be seen in perfectly coordinated gait. Bottom right shows the opposite, an uncoordinated pattern of two simulated random time series with negligible diagonal lines.

Based on the model of optimal movement variability (Stergiou et al., 2006), a theoretical model of coordination can be considered as shown in Figure 1. In this model, we see two extremes of coordinative states that exhibit either metronomic qualities or random qualities. Both these states can be indicative of unhealthy pathological disease processes. Healthy inter-limb coordination sits at a sweet-spot between these systems. Healthy natural gait is characterized by two limbs effectively working together providing smooth, flexible, and efficient gait (Combs et al., 2013). In disease processes like stroke or Parkinson’s disease, this optimal coordination is affected (Roemmich et al., 2013; Arya and Pandian, 2014).

For example, asymmetric pathology, like stroke can cause people to display larger between leg phase-shifts in response to a perturbation compared to healthy controls (Krasovsky et al., 2013). Inter-limb phasing using cross-correlation showed characteristic adaptive changes in bilateral coordination tasks like split-belt adaptation in stroke survivors (Reisman et al., 2005; Reisman et al., 2007). In other neurological conditions, like Parkinson’s disease, was shown in comparison to reduced inter-limb coordination compared to healthy controls (Plotnik et al., 2007; Plotnik et al., 2008; Roemmich et al., 2013). A traditional measure, phase coordination index, was significantly increased in people with Parkinson’s disease compared to a healthy sample, indicating worse bilateral coordination (Plotnik et al., 2007; Plotnik et al., 2008). Another coordination variable, cross-covariance was utilized to show that ipsilateral and contralateral inter-limb coordination was reduced in people with Parkinson’s disease (Roemmich et al., 2013). For such patient populations, who have been affected by neurological diseases, an exoskeletal system such as a wearable robotic gait-assistive device, may improve inter-limb coordination.

### 1.2 Exoskeletal Assistance as an Approach to Restore Distorted Gait

For many years, researchers have shown how active (powered) and passive (powered) exoskeletons affect the way people perform different walking tasks (Takahashi et al., 2015; Galle et al., 2017; Kim et al., 2019; Awad et al., 2020). In the field of exoskeleton research, most of the effort has been directed towards the reduction of energy expenditure and muscle activations, and these studies have revealed the mechanical benefits of exoskeleton assistance (Collins et al., 2015; Awad et al., 2017; Kim et al., 2019). However, it is still not known how such assistive devices change inter-limb coordination patterns during walking. In this study, a passive exoskeleton was used. This device (Glaister et al., 2015) was originally built for patients like stroke survivors or spinal cord injury. In brief, this device has a cable that runs through the ankle, knee and hip joints and is attached to a spring. During the stance phase of walking, the cable pulls the spring, stores energy, and returns the energy as the toe propels forward to help swing the leg forward (Glaister et al., 2015).

Exoskeleton assistance can potentially alter inter-limb coordination by making gait either 1) more periodic, i.e., like a sinusoidal wave or 2) more random. It is also possible that exoskeleton-assisted gait may show no change in coordination in comparison to walking without such assistance. While healthy young individuals can produce somewhat periodic, but complex fluctuations (Stergiou et al., 2006), it is not well understood how exoskeleton assistance affects inter-limb coordination. Therefore, the purpose of this study was to determine which specific direction exoskeleton-assisted gait would take inter-limb coordination patterns toward – either a more periodic or a more random pattern as shown as in Figure 1. The clinical implication of such an alteration would inform the development of devices that can restore healthy inter-limb coordination in patients.

### 1.3 Coordination Measurements

To quantify coordination, traditional coordination measurements include discrete relative phase (Kelso et al., 1992), continuous relative phase (Lamb and Stöckl, 2014), cross-correlation (Park et al., 2012), and normalized root mean squared difference (Loiret et al., 2019). However, each of these methods have certain limitations which prevent a complete picture of the complex dynamic nature of gait coordination from emerging. These measurements provide discrete outcomes based on the time series which only look at *snapshots* of movements. However, human movements are rather dynamic and continuous and are like *movies*. These linear measurements may be sufficient to describe some parts of dynamical systems, but it may not tell the whole story. This is of critical importance in special populations, like stroke and unilateral amputees, where one half of the body is impaired by the disease with characteristic effects distinctly different from the other half. Therefore, in these situations, the overall effect is one of an abnormally coordinated multi-segment unit. In addition, both discrete relative phase and continuous relative phase are based on the assumption that the two limbs during gait, are at a one-to-one frequency, or completely periodic like a sine wave (Peters et al., 2003). Unless appropriate variables are used to assess such abnormal behavior, the impact on therapy and rehabilitation may not be significant. Examples of such assessment instruments are cross recurrence quantification analysis (cRQA) and cross sample entropy (cSE). These nonlinear methods of analyses can capture the evolution of the movements and quantify the coordination between the left and right legs.

The cRQA technique is a modified version of recurrence quantification analysis (RQA) and quantifies how long two different dynamical systems exhibit coordinated patterns of behavior over time (Coco and Dale, 2014). In this analytical method a recurrence plot is created as a result of reducing a higher-dimensional, nonlinear pattern into two-dimensions, which shows the recurring patterns or trajectories of that dynamical system (McCamley et al., 2017). Changes in physiological or environmental dynamics alter the duration of coordination (McCamley et al., 2017; Dutt-Mazumder et al., 2018), in effect quantifying the three aspects of our theoretical model in Figure 1.

Sample entropy is a nonlinear analysis method which quantifies the regularity or predictability of a single time series (Richman and Moorman, 2000; Lake et al., 2002). Larger sample entropy values indicate more irregularity in a time series. While sample entropy examines the regularity of a single time series, cSE measures the synchrony between two time series. In other words, it examines whether there are similar patterns between two data series (McCamley et al., 2017) with larger cSE values indicating more asynchrony.

### 1.4 Hypotheses

The focus in our study was to determine how inter-limb coordination was affected through exoskeleton-assisted gait. It was hypothesized that imposed passive exoskeleton assistance would lead to a change in the dynamics of inter-limb coordination. This will be demonstrated with a reduction in duration of coordination and synchrony of gait patterns without changing conventional gait measurements like step length, step time, step width, and walking speed. Specifically, we hypothesized that inter-limb coordination would show characteristic decreases in coordination duration and synchrony when walking at preferred walking speed (PWS) with a unilateral exoskeleton device if the device helped to make gait more explorative. Alternatively, these two measures would be flipped, i.e., increased duration and synchrony, if exoskeletal-assisted gait resulted in increased restriction.

## 2 Methodology

*Participants:* In this study, 18 young healthy individuals (19–35 years) were recruited. Recruited participants did not have any lower limb dysfunction, cognitive or neurological impairments, nor vascular or other abnormalities that could affect walking on a treadmill. All participants were right leg dominant and had previous exposure to treadmill walking. No participants were excluded. Prior to the experiment, each participant signed an informed consent approved by the Institutional Review Board from the University of Nebraska Medical Center. All procedures were approved by, and conducted according to the guidelines of the University of Nebraska Medical Center Institutional Review Board.

*Research design*: Participants were randomly placed into either a group that walked with the passive exoskeletal device (EXO) or a group that walked without the exoskeleton device (NO EXO). To select their PWS, all participants walked at a self-selected comfortable speed, similar to normal, everyday walking. Speed was increased gradually as the participants walked on the treadmill and participants were instructed to let the examiner know when PWS was reached. All participants first walked on the treadmill for a familiarization trial for 5 minutes. Then, for those participants who were placed in the EXO group, the device was attached to their right dominant leg. The dominance of the leg was determined by asking each participant which leg they would use to kick a ball. After the exoskeleton was attached, participants confirmed if their PWS was comfortable or it needed to be changed. None of the participants requested to change their speed. Participants in the EXO group had an extra familiarization trial where they walked on the treadmill for 5 minutes while wearing the passive exoskeleton.

To collect experimental data, all participants were asked to walk for 5 minutes at their PWS. Movements of the participants were captured using a digital motion capture system at 100 Hz (8-camera T160, Vicon, Oxford, United Kingdom). Participants wore tight-fitting suits to avoid capturing the movement of clothing. Each participant had 11 markers attached to specific anatomical landmarks that included the anterior superior iliac spine, posterior superior iliac spine, sacrum, lateral malleolus, head of the second metatarsal, and the calcaneus (Mukherjee et al., 2015).

**FIGURE 2:**
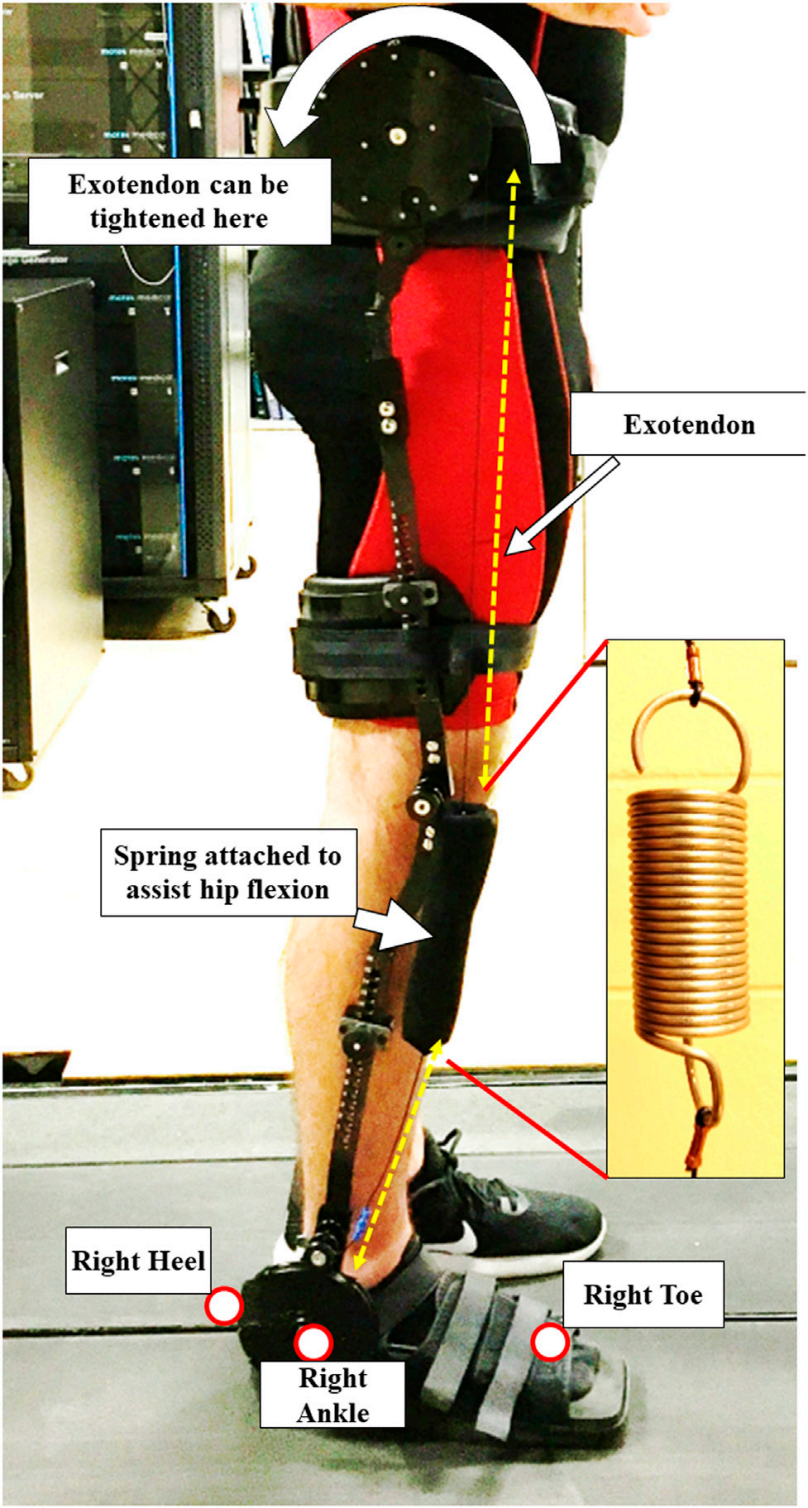
A participant with the passive exoskeleton attached to their right dominant leg. The spring is attached to a cable called the Exotendon, which can be tightened by the disk on the waist. While a person is in the stance phase of gait, the spring is extended, storing energy. When a person propels forward, the spring provides assistance by returning the energy. The figure also shows the positions of the retro-reflective markers that were used in the study.

*Exoskeleton:* The exoskeleton device, used in this project, was the Kickstart™ Walking System (Cadence Biomedical, Seattle, WA). This passive device (Figure 2), which weighs approximately 7.5 pounds (3.402 kg) (Sado et al., 2022), used an elastic cable (Exotendon™) (Glaister et al., 2015) inspired by a biomechanical model (van den Bogert, 2003). The cable runs parallel to the lateral side of the leg and through pulleys over the three joints (ankle, knee, and hip). The knee joint of the exoskeleton was aligned to the participants’ medial-lateral rotational axis at their knee. The exoskeleton was fitted with special shoes, similar to a boot one would wear after breaking a toe (Figure 2). The exoskeletal device was adjusted for each participant at their waist, thigh, and shank. The device was firmly fitted without causing any physical discomfort to the participants. Tightness of the cable was adjusted using a disk located on the side of the waist belt. The cable was first tightened to the point where all slack was taken up, then it was tightened until participants felt the assist. This was confirmed by having participants walk overground with the device for a minute.

*Data analysis*: All data processing was done with custom code (Matlab 2019b; Mathworks, Natick, MA). Step length (m), step time (sec), and step width (m) were measured as basic outcomes of performance. Step length was defined as the distance from the heel-strike to contralateral heel-strike in the anterior-posterior direction. Treadmill belt movement was added to calculate step length. Step time was calculated for each side as the duration of time between the contralateral heel strike to the ipsilateral heel strike. Step width was defined as the mediolateral distance between heel markers at successive heel strikes. For each leg, right and left, the first 200 steps were averaged for each of the spatiotemporal outcomes.

**FIGURE 3:**
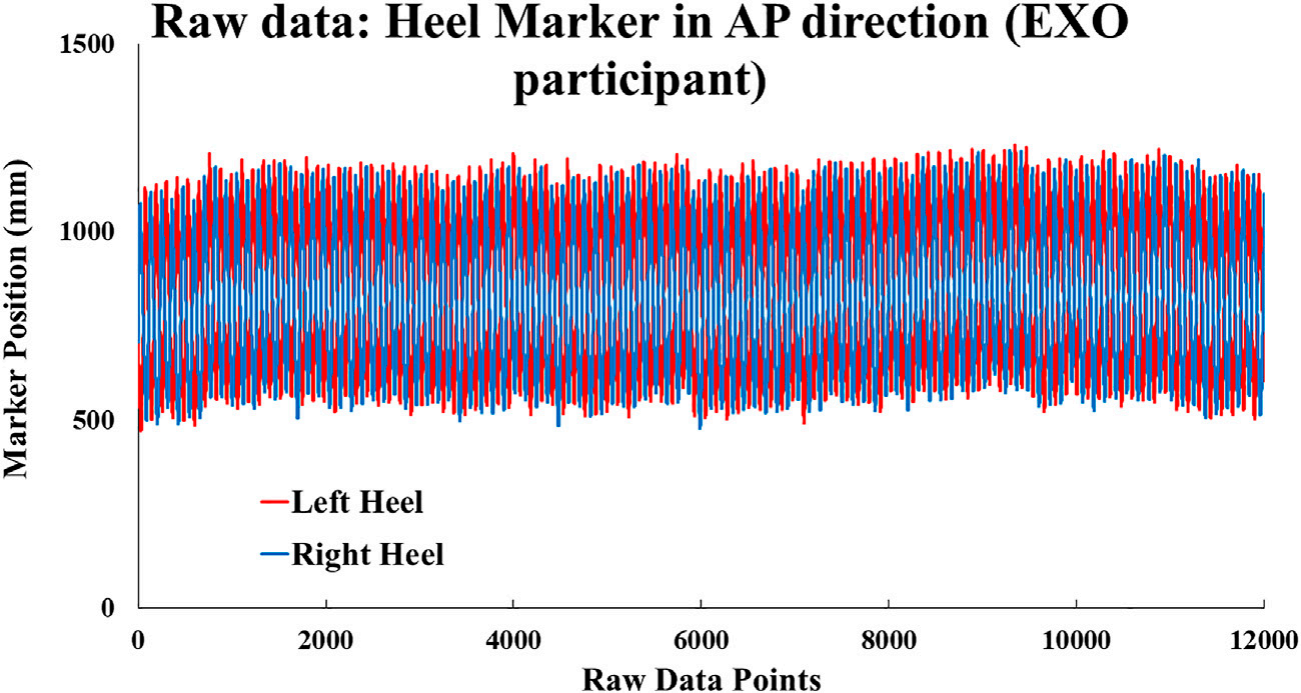
Raw position data in mm of a representative participant’s right and left heel markers in the AP direction.

The raw marker data from the left and right heel (Figure 3) sagittal plane (anterior-posterior direction) were used for analysis of cRQA and cSE (*The custom code is available in the supplemental section of McCamley et al 2017, doi: 10.1155/2017/7960467*) (McCamley et al., 2017). Specifically, 12,000 data points were down sampled by 10 Hz to 1,200 data points for further analysis. Data was not filtered.

As part of cRQA, state-space was reconstructed for each pair of right and left heel marker data. Time delay (τ, tau) was calculated via average mutual information method (Hanchuan Peng et al., 2005) for each leg. The first minimum tau value was used. Optimal embedding dimension was computed with the false nearest neighbor analysis (Kennel et al., 1992). Embedding dimension for each participant and leg were calculated separately (Table 1), and the maximum embedding dimension was recorded. When the embedding dimensions of the two legs were different, the higher value was used.

**TABLE 1:** Time delay and the EMB values for each participant in the EXO and the NO EXO groups. These values were used to reconstruct state space figures and perform cRQA.

Participant ID	Time Delay (τ)	EMB	Participant ID	Time Delay (τ)	EMB
NOEXO04	3	5	EXO01	3	5
NOEXO08	3	5	EXO02	3	6
NOEXO09	3	5	EXO03	3	5
NOEXO10	3	5	EXO04	3	5
NOEXO13	3	4	EXO10	4	5
NOEXO15	3	5	EXO11	3	5
NOEXO16	3	5	EXO13	3	5
NOEXO21	3	5	EXO14	4	6
NOEXO32	3	5	EXO17	3	5

Using the computed τ and embedding dimension, right and left heel markers were reconstructed into their state space. To determine if the time series were recurrent in the same state space, a tolerance window (i.e. radius) was used. The size of the radius fluctuated between participants to ensure a 2.5% recurrence rate. The recurrence rate was selected based on the assumption that sparse recurrence plots with scattered points reveal the most information (Zbilut and Webber, 1992; Shockley et al., 2002; Wallot, 2017). The resultant output was a two-dimensional recurrence plot. Each point on the recurrence plot represents a point in time the movement between the two legs were coordinated. Once the recurrence plot was constructed, the length of diagonal lines, defined as a minimum of two recurrent points in length, were quantified. The following cRQA features of the recurrence plot were extracted to inform the characteristic dynamics of the system.

1) *Embedding Dimension* (EMB): A time-series needs to be unfolded to the maximum embedding dimension (Zbilut and Webber, 1992) to ensure that all meaningful dynamics are observed. No new information is observed beyond the determined EMB. If EMB is similar between groups, the dimensionality required to provide an accurate assessment of coordination behavior is not different between the groups. Biologically speaking, it would mean that both groups demonstrate optimal inter-limb relationships at similar dimensionality with no new information added if dimensionality is increased.
2) *Radius*: the tolerance or cut-off value for the distance between the trajectory of one system (e.g., left leg) and another system (e.g., right leg) to be counted as recurrent. To maintain the recurrence rate of 2.5%, radius fluctuated from participant to participant. A larger radius indicated the limb trajectories were further apart (increased interaction between the limbs) and when smaller, the trajectories were closer together (reduced interaction).
3) *Percent determinism* (%DET): was the percentage of recurrent points falling on a diagonal line compared to all points on the plot. This value is interpreted as the determinism of how two signals are coordinated. A %DET closer to 100% indicated an almost perfectly coordinated system.
4) *Mean Diagonal Line Length* (MeanL): was the average length of the diagonal recurrence lines and indicated the average time that the two signals spent in the same region of the reconstructed state space.

Cross sample entropy (cSE) was the probability that patterns in one time series will appear in the other time series. cSE used input parameters of the length of data, vector length, and tolerance. Specific calculation of the cSE can be found elsewhere (Richman and Moorman, 2000). In brief, it divided the two different time series with the same length into short vectors of length *m*. For each of these vectors, it compared a vector from one time series to a vector from the other time series to determine whether the vectors were similar. Two vectors were considered similar if they were within the tolerance, *r*. This process was repeated in steps with vectors one longer than m (i.e. *m*+1). Based on the recommendations from McCamley et al. (2017), vector length, *m*, was set to 3. Tolerance, the allowable region of data to be considered a match in patterns, was set to 0.25 * standard deviation.

In total, the dependent variables subjected to analysis were step length, step time, and step width; cRQA EMB, radius, %DET, MeanL; and cSE. All dependent variables were examined for normality using a Shapiro-Wilk test. EMB failed the normality check. Step length R for EXO and step length L for NO EXO failed the test of normality distribution as well. Transformation of data did not resolve the normality violation. Therefore, we performed Mann-Whitney U nonparametric tests for EMB and step length (left and right). Statistical differences for rest of the measurements (%DET, MeanL, radius, and cSE) were examined via independent t-tests between EXO and NO EXO. For all statistical tests, alpha level was set at 0.05. Statistical analysis was done using SPSS (version 26, IBM Inc., Armonk, NY).

## 3 Results

Participant demographics are shown in Table 2. There were no significant differences between the groups for anthropometric measures. The two groups did not differ in their PWS. The mean and standard deviation of spatial and temporal measures showed no significant differences between the two groups during treadmill walking at their PWS (Table 3). Mann-Whitney U test showed no difference between the groups for EMB and step length.

**TABLE 2:** Participant demographics. The two groups were not different in any of the demographics or measured walking speeds. PWS = preferred walking speed.

Independent t-test: N = 18 (EXO: N = 9/NO EXO: N = 9)				T-test
	Group	Mean	SD	*p*-value
Age (years old)	EXO	23.2	3.0	0.424
	NO EXO	24.2	2.1	
Height (cm)	EXO	177.6	5.9	0.767
	NO EXO	178.6	8.4	
Mass (kg)	EXO	79.8	6.8	0.974
	NO EXO	79.9	14.0	
PWS (m/s)	EXO	1.12	0.15	0.734
	NO EXO	1.10	0.10	

**TABLE 3:** Mean of the first 200 steps for the linear measurements in each limb for the two groups. There were no significant differences between the EXO and NO EXO groups.

Independent t-test				
	Group	Average	SD	*p*-value
Step Length R (m)	EXO	0.59	0.06	0.35
	NO EXO	0.57	0.03	
Step Length L (m)	EXO	0.59	0.06	0.71
	NO EXO	0.58	0.04	
Step Time R (sec)	EXO	0.61	0.05	0.07
	NO EXO	0.58	0.03	
Step Time L (sec)	EXO	0.57	0.04	0.79
	NO EXO	0.58	0.03	
Step Width R (m)	EXO	0.17	0.02	0.47
	NO EXO	0.16	0.03	
Step Width L (m)	EXO	0.17	0.02	0.47
	NO EXO	0.16	0.03	

**FIGURE 4:**
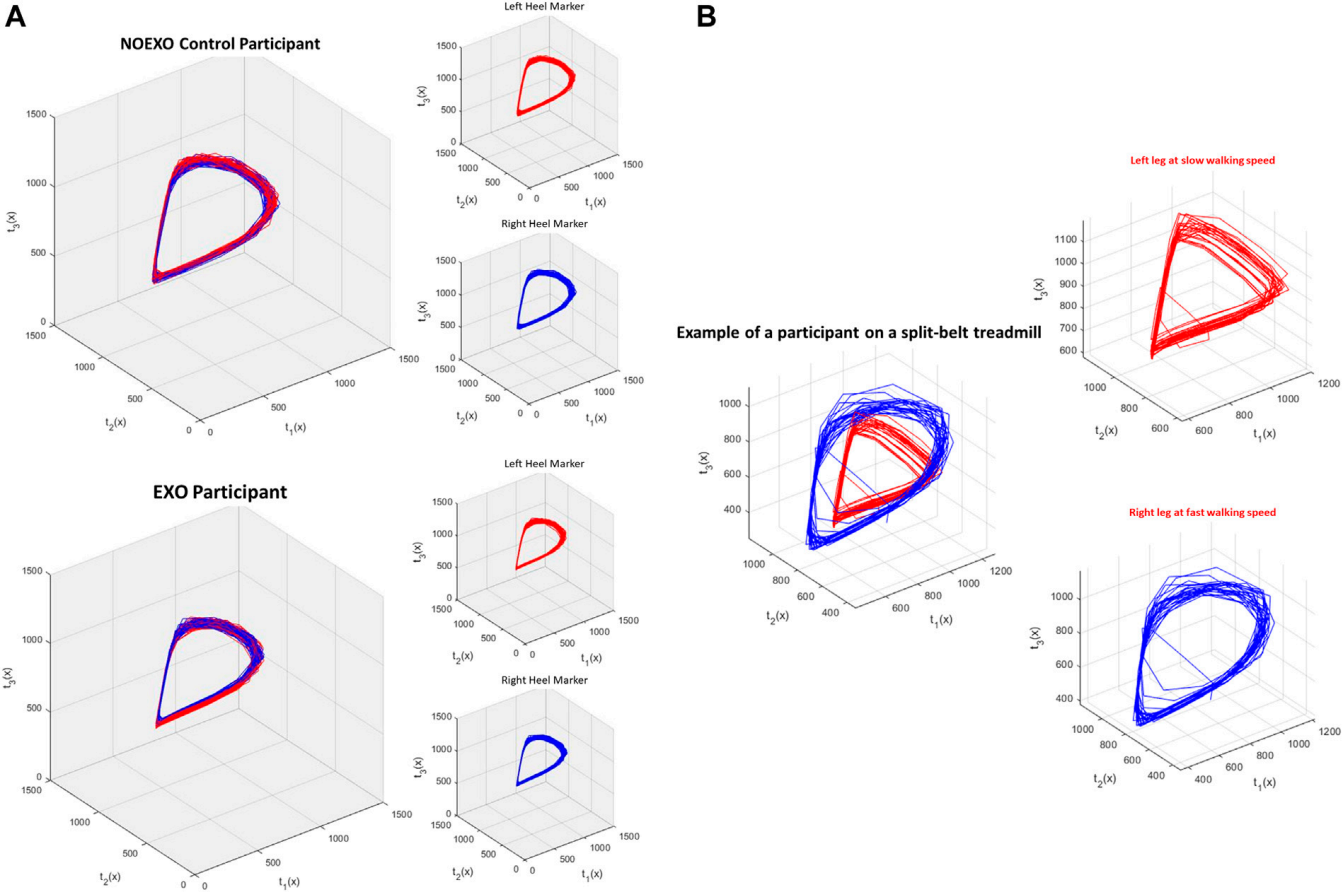
**(A)** and **(B)** shows the state space reconstruction figures from representative participants. Each set of three figures show state space reconstruction for left heel marker movement (red), right heel marker movement (blue), and combined. Axis t_1_(x) shows the original time series of heel marker movement. Based on the time delay that was found, axis t_2_(x) and t_3_(x) were created [e.g. if time delay was 3, t_2_(x) would be t_1_ (x + 3) and t_3_(x) would t_2_ (x + 3)]. **(A)** top shows the reconstruction figure of a participant walking without an exoskeleton (NO EXO), and **(A)** bottom shows the reconstruction figure of a participant with the exoskeleton (EXO). For both participants, the overall gait dynamics in each leg was similar, showing a teardrop like shape. This indicates that even with exoskeleton assistance, the normal cyclical, rhythmic pattern in each leg was not impacted. **(B)** shows the state space reconstruction figure of a participant performing a split belt adaptation task. This is the same EXO participant from **(A)** performing a different study. During the split belt task, the left leg was slower than the right, altering the reconstructed state space.

**FIGURE 5:**
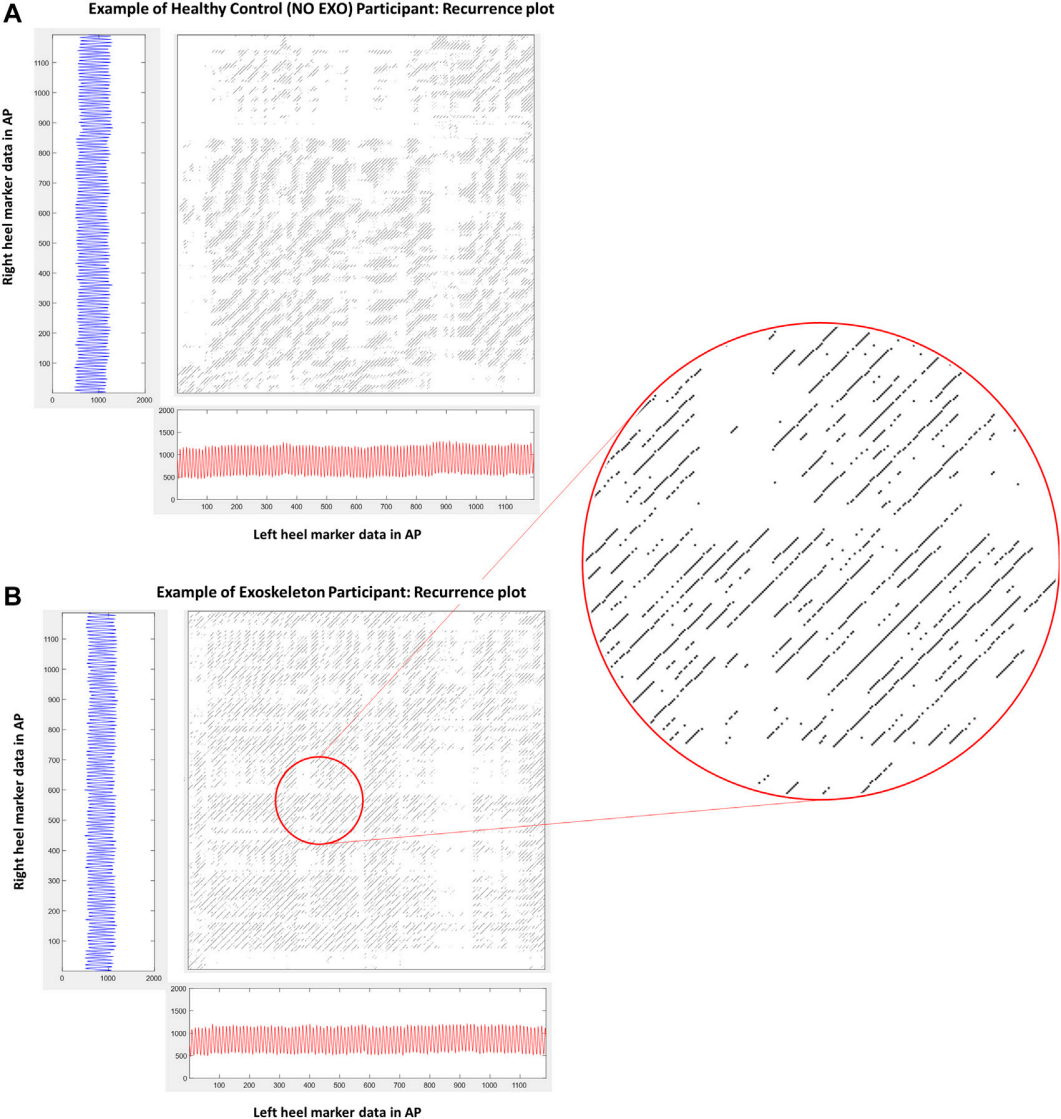
**(A)** and **(B)** shows the recurrence plot of one participant from each group. These are the same participants as in Figure 4. **(A)** shows the recurrence plot of a participant walking without an exoskeleton (NO EXO). **(B)** shows the recurrence plot of a participant walking with exoskeleton assistance (EXO). The inset figure is a zoomed in section showing diagonals on the recurrence plot whose length characterize the duration of inter-limb coordination while synchrony is characterized by the presence of similar patterns within the plot.

Analysis of inter-limb coordination patterns through cRQA and cSE showed significant group differences. State-space reconstruction figures for one participant performing the treadmill walking task without the exoskeleton (Figure 4A) and another participant performing the task with the exoskeleton (Figure 4B) shows the characteristic evolution of each heel marker trajectory over time. Recurrence plots are presented for one participant performing the treadmill walking without the exoskeleton (Figure 5A) and another participant performing the task with the exoskeleton (Figure 5B).

**FIGURE 6:**
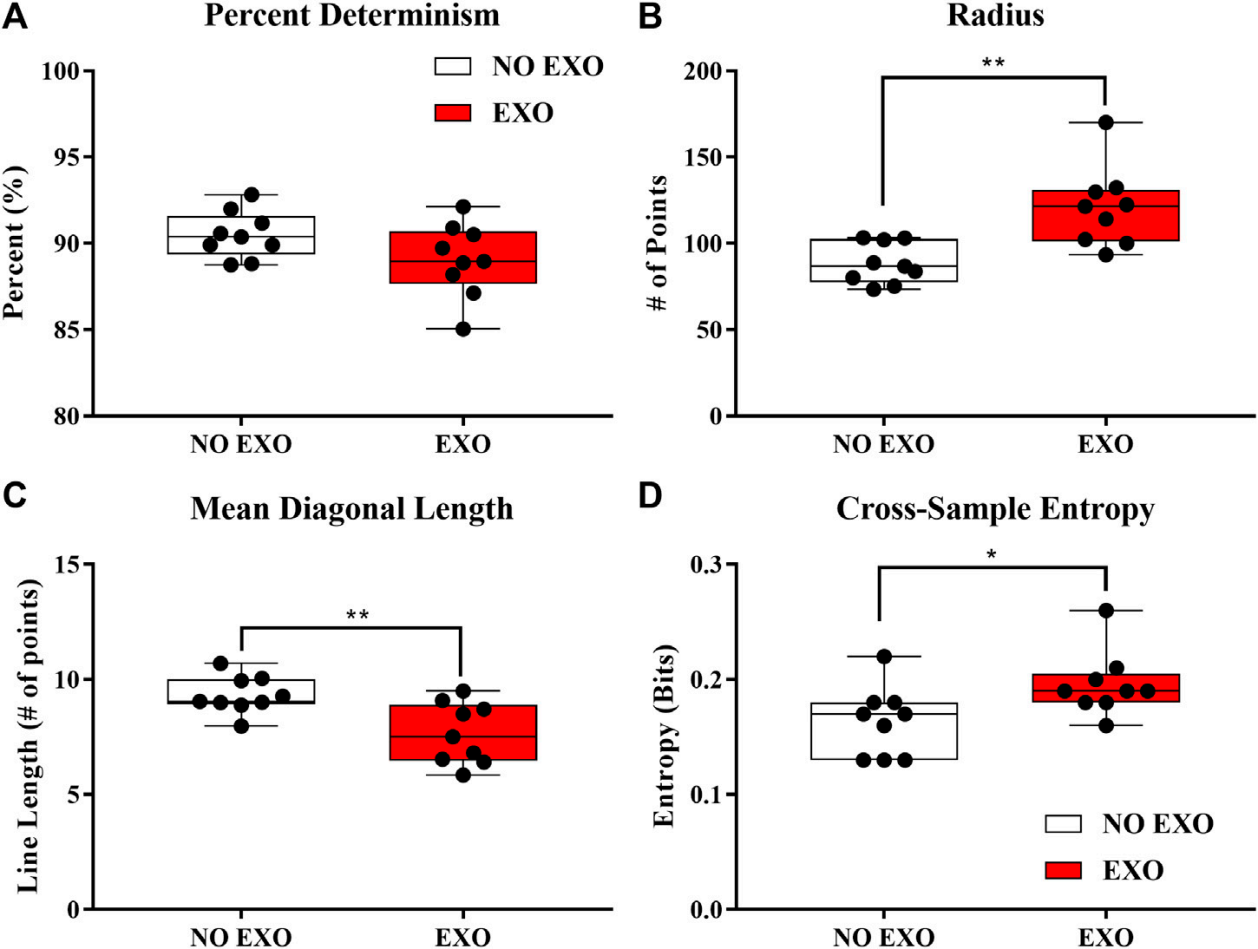
**(A–D)** Box and whisker plots showing inter-quartile ranges. **(A)** shows the main cRQA variables that were analyzed. **(B)** shows the entropy value from cSE. Each dot represents one participant. Significant differences between the EXO and the NO EXO groups are shown. ∗*p < 0.05*, ∗∗*p < 0.01*, and ∗∗∗*p < 0.001*.

Significant group differences in the cRQA parameters (%DET, Radius, and MeanL) are shown in Figure 6. Specifically, there was no difference in EMB (*p* = 0.176). This means that the dimensionality (information) required to provide an accurate assessment of coordination behavior is not different between the groups. Radius was significantly larger [t (16) = 3.741, *p* = 0.002] in the EXO group (120.64 ± 22.91 points) compared to the NO EXO group (88.49 ± 11.82 points). This means that, for EXO group, a larger radius was required to incorporate the same number of recurrent points as the NO EXO group. This may be an indication of reduced interactions between the limbs. Percent determinism (%DET) did not show significant difference between groups (*p* = 0.357). No difference in %DET suggests that wearing an exoskeleton does not change the determinism of coordination between the two limbs compared to the NO EXO group. Mean diagonal length (Mean L) was significantly shorter [t (16) = −3.228, *p* = 0.005) in the EXO group (7.65 ± 1.33 points) compared to the NO EXO group (9.31 ± 0.80 points), which indicates that average duration time that the two limbs were coordinated was shorter in the EXO group.

The cSE measure was shown to have a significantly higher value [t (16) = 2.552, *p* = 0.021] in the EXO group (0.20 ± 0.026 bits) compared to the NO EXO group (0.16 ± 0.03 bits), indicating increased asynchrony of heel trajectories between the right and left feet when walking with the exoskeletal device compared to natural gait.

## 4 Discussion

The purpose of the current study was to examine how passive assistance, provided by an exoskeletal device would lead to a change in the dynamics of inter-limb coordination, which could be demonstrated with a reduction in the duration of coordination and the synchrony of gait. Our results provided evidence in favor of both primary hypotheses that walking with the device showed inter-limb coordination patterns that were of shorter duration and lower regularity than walking without the device. This indicated that assistance from the exoskeleton had potential to make gait more exploratory rather than more restricted. Further, this was achieved without any associated differences in the linear measures (Table 2).

### 4.1 Passive Exoskeleton Does Not Disrupt Spatiotemporal Gait Patterns

No differences were found in average step length, width, and time between the two groups. The participants, walking at their PWS, adapted to the exoskeleton without requiring spatio-temporal adjustments. Walking with a unilateral passive spring-loaded exoskeleton was not a complicated task. Exoskeleton studies in healthy (Malcolm et al., 2013; Galle et al., 2017) and pathological populations (Glaister et al., 2015; Awad et al., 2020) have not shown changes in gait patterns during treadmill walking. Although the design of exoskeletons with unilateral or bilateral placement may intuitively be expected to have effects on step width, typically it has been found that step width is less likely to be affected by the exoskeleton (Henderson et al., 2017; Lee et al., 2017). This may also be due to the requirement of staying at the center of the treadmill (both anteroposterior and mediolaterally) at constant speed.

In our work with stroke survivors, we found that walking with this device for 5 days changed spatiotemporal patterns, specifically step time, length and width and double support time (Yao et al., 2021). The differences in the results here in comparison to that study could be due to healthy participants in this study who would be generally more adaptive (Sado et al., 2022), had less exposure to the EXO device, and were walking on a treadmill instead of over ground.

### 4.2 Determinism and Dimensionality are Not Affected by Passive Assistance During Gait

Determination of the EMB is an important step towards reconstructing the time series to maximize the information that can be extracted from a complex nonlinear system. This can be further visualized through state space reconstruction (Figure 4). Walking with the device did not change how many dimensions were needed to maximize the information extracted from the dynamical system. In healthy young individuals, state-space reconstruction of normal treadmill walking showed a tear-drop shape (Figure 4A), and this structure was maintained even when people walked with the exoskeleton (Figure 4B).

The radius, however, was larger in the EXO group. To assess the coordination duration between the limbs, a larger radius was needed while the recurrence rate was kept to 2.5%. This means that the two trajectories were further apart for the EXO group. It can be speculated that inter-limb coordination has certain constraints that makes it healthy. A passive exoskeleton assistance may remove some of these constraints, making people more explorative. However, this does not mean that the smaller radius causes the duration of coordination to be shorter.

There were no differences in the %DET between the EXO and the NO EXO group. If coordination patterns were highly deterministic as in robotic gait, %DET would have been close to 100%. In this study, we found that %DET was approximately 90% in both groups, demonstrating that inter-limb coordination in healthy young participants was characterized by a high degree of determinism. Specifically, the chances of finding recurrent points forming diagonal lines in the recurrence plot was high, around 90%. A similar level of determinism has been shown in healthy humans when coordination between different physiological systems such as breathing and walking were considered (McCamley et al., 2017). When the sensorimotor systems are affected, such as in Parkinson’s disease (Afsar et al., 2018) and hypovestibular disorders (Sylos Labini et al., 2012), recurrence patterns were demonstrated to be less deterministic in comparison to healthy. In postural tasks, %DET has been shown to be consistently high in healthy participants and were affected by specific tasks such as maintaining balance during high frequency oscillations of the support surface (Dutt-Mazumder et al., 2018). In that study, %DET was reduced significantly only when oscillations crossed a specific threshold and became too difficult. In our study, it was shown that addition of a unilateral exoskeletal device did not affect the determinism of recurrence points on the diagonal lines. Therefore, our task of walking at PWS being a simple task and the participants being healthy, essentially led to both groups being similarly deterministic.

### 4.3 Walking with a Passive Exoskeleton Changes the Duration of Inter-Limb Coordination

Walking with the exoskeleton led to a reduced average time that the two limbs were coordinated at PWS compared to those that did not wear the exoskeleton. Theoretically, robotic gait is likely to produce very long mean diagonal lengths because each limb produces periodic sinusoidal movements that repeat perfectly. On the contrary, there is an inherent variability in healthy human dynamical systems (Mukherjee and Yentes, 2018) and such variability is characterized in the recurrence plots (Figure 5A). When healthy young individuals walked with a unilateral limb load, their inter- and intra-limb coordination (continuous relative phase and cross correlation) were affected such that inter-limb coordination measures were reduced (Peters et al., 2003). In our theoretical model, duration of coordination was reduced when walking with the device. Specifically, in Figure 1, exoskeletal-assisted gait guided the system towards the right side of the model. This was possibly due to reduced coordination between the limbs allowing for greater exploration, instead of tighter coordination that potentially restricted adaptive flexibility. Alternatively, it is possible that the weight of the exoskeleton influenced the reduction in coordination and teasing out the effect of the device weight would require further investigations.

In pathology such as chronic obstructive pulmonary disease (McCamley et al., 2017), coupling between physiological time series such as breathing and walking became more rigid with longer mean lengths in comparison to healthy participants. This was believed to indicate a reduced coupling between physiological systems in healthy participants allowing for greater variability and essentially stemming from a need to be more flexible and adaptable. Intuitively, the changes induced with the passive exoskeleton maybe an indication of more flexible and adaptive behavior that such an artificial system allows. This may provide a window of opportunity to make unhealthy, restrictive gait such as in stroke, more adaptive and flexible with the use of exoskeletal devices.

### 4.4 Passive Exoskeleton-Assisted Treadmill Walking Reduces the Synchrony of Inter-Limb Coupling

Exoskeleton-assistance reduced the synchrony between the two limbs during walking at PWS. Jordan et al. (2006) showed that when people walk at PWS, they have the lowest α-value (using detrended fluctuation analysis) in comparison to non-preferred speeds (higher/lower than PWS) where strength of long-range correlations increase (Jordan et al., 2006). This has been understood to be the result of an increase in dynamical constraints and therefore, a reduction in the available degrees of freedom as we move away from PWS. In our study, it appeared that adding a spring-loaded, unilateral, passive device enabled the participants to increase these degrees of freedom and walk with a greater explorative ability. Similar to the addition of an exoskeleton, adding sensory stimuli such as vibratory insole tactors (Chien et al., 2017) or auditory feedback (Kaipust et al., 2012; Hunt et al., 2014) changed dynamical patterns during walking. Taking these results further, we have shown that adding assistive constraints led to changes in inter-limb coordination dynamics specifically to the synchrony of these coordination patterns such that walking became less repeatable and more adaptive.

### 4.5 Reduced Inter-limb Coordination for Explorative and Flexible Gait Patterns

In our previous research, we investigated how healthy young individuals utilize the exoskeleton assistance to learn a novel walking task (Sado et al., 2022). In that study, we used a split-belt adaptation paradigm (Mukherjee et al., 2015) where one leg moved at a faster speed than the other leg because they were on treadmill belts with different speeds. It was found that in comparison to controls, exoskeleton assistance helped with motor adaptation tasks, specifically for the stance time during the early and late learning phases. Additionally, it was found that the center-of-mass (COM) positive work became more symmetric in comparison to controls during the adaptation task. This is important because the control group also adapted to the task by reducing COM positive work asymmetry. If the exoskeleton assistance was restricting walking, it would also interfere with the ability to adapt to a novel gait task. The increased COM positive work symmetry during exoskeleton use may have allowed gait patterns to be more flexible and adaptive. In this current study, we show that this adaptive change may have been achieved through the optimization of gait coordination dynamics i.e., by reducing duration and synchrony of inter-limb coupling. Since higher duration and synchrony are indicators of restricted gait, we speculate that passive exoskeleton-assistance makes gait patterns more explorative to allow greater adaptive flexibility.

### 4.6 Relevance/Clinical Impact

Our findings indicated that a passive exoskeleton induced more exploration by changing the coordination between the limbs. Such changes could be very important for those patients whose movements are constricted. A passive exoskeleton reducing the recurrent gait patterns (less duration and synchrony) could allow error-based learning (Bastian, 2008; Lewek et al., 2018). This means that exoskeleton assistance may remove coordination constraints, allowing the individual to explore the task and environment more, so that error-based learning can occur. In many pathological cases such as those with neurological deficits or sensorimotor deficits, abnormal coordination has been shown (Plotnik et al., 2008; Meijer et al., 2011; Williams et al., 2013). A passive exoskeleton has the potential to break such restrictive abnormal coordination patterns, allowing patients to restore healthy inter-limb coordination. Future work could assess such dynamics during inter-limb coordination tasks like split-belt adaptation.

### 4.7 Limitations and Future Directions

In this study, some limitations were identified. One of the limitations was the between-subjects design. A within-subjects approach may provide more information about change in intra-person behaviors. Although it appears that everyone adapted to the device, there were no measurements of adaptation. Within-subjects test may also show how a person adapts to an assistive device. Also, treadmill walking is known to alter gait compared to overground walking (Lee and Hidler, 2008). Examining inter-limb coordination during overground walking using nonlinear tools may reveal different effects of exoskeleton assistance.

For the exoskeleton that was used for this study, special shoes had to be worn, which may have affected the way people walk. However, all participants were given a 5-min familiarization trial to get adjusted to walking with this device. Additionally, there was no objective way to know the tightness of the tendon. In related studies (Yao et al., 2021; Sado et al., 2022), the number of ratchet clicks on the disc at the waist was used to have a generic measure of the exotendon tightness. The EXO group only had an exoskeleton on one side. Moreover, it is still unknown whether the reduced duration and synchrony of inter-limb coordination was due to the weight of the device or asymmetry between the limbs. To answer these questions, in a future study we are focusing on how unilateral and bilateral limb loading changes the specific coordination measurements.

## 5 Conclusion

The current investigation focused on how passive exoskeleton assistance affected the duration and the synchrony of inter-limb gait coordination. It was found that walking with passive assistance decreased the duration and the synchrony of coordination between the limbs. This could indicate that patient populations with abnormal inter-limb coordination could utilize such assistive devices, the assistance could disrupt the abnormal coordination patterns between the limbs, allowing the wearers to explore the environment, and possibly help them restore healthy coordination.

## Data Availability

The datasets generated during and/or analyzed during the current study are available from the corresponding author on reasonable request.
